# The Select Medical Library and Eclectic Journal of Medicine

**Published:** 1840-07

**Authors:** 


### BIBLIOGRAPHY.

*The Select Medical Library and Eclectic Journal of Medicine, edited by* John Bell, M. D., *Lecturer on the Institutes of Medicine and Medical Jurisprudence ; Member of the College of Physicians of Philadelphia, and of the Amer. Philos. Soc., etc. Vol. III, No.* 10,—*August,* 1839.—*Philadelphia, Haswell, Barrington & Haswell.—pp.* 240.

The above work is published monthly ; each number contains 240 pages—thirty-six of which are devoted to the latest medical intelligence and bibliographical notices of new medical works ;—the balance, 204 pages, to the re-publication of standard works on medicine.

Among medical men, this work is too extensively and favorably known to need any commendation from us. The distinguished ability with which it is conducted, has gained for it, a justly deserved, high reputation. We would, however, invite more particularly the attention of the members of our own immediate profession to it, feeling assured that they would not only derive interest, but also much advantage from the perusal of its pages. The importance of a more general and extensive knowledge of medicine than is possessed by a large proportion of the profession, is now universally conceded; but we forbear to enlarge upon the subject here, having spoken upon it somewhat at length in another part of the present number of this journal, to which we would refer the reader.

The works that have been re-printed in " The Select Medical Library," are all valuable, and should be possessed by, not only every member of the medical, but also those of the dental profession. Among them are those of the celebrated John Hunter, including his " *Treatise on the Natural History and Diseases of the Human Teeth*," with notes by Thomas Bell, F. R. S., &c. &c., which is contained in the August number, and which, without Mr. Bell's notes, is now being published in " The American Journal of Dental Science," with notes by the New-York editor. The works of Mr. Hunter, of themselves are worth at least two years' subscription to the Library, for with the exception of this edition they are very scarce.

The August number of the " Eclectic Journal of Medicine," contains a very interesting case of Catalepsy, taken from the Southern Literary Messenger, which, on account of the connexion it seems to have had with a carious tooth, we should like to have copied into our own pages, but are prevented from doing so, in consequence of its great length. We may, however, present it to our readers in some subsequent number.

[BALT. ED.

